# A quasi-Newton algorithm for large-scale nonlinear equations

**DOI:** 10.1186/s13660-017-1301-7

**Published:** 2017-02-03

**Authors:** Linghua Huang

**Affiliations:** School of Information and Statistics, Guangxi University of Finance & Economics, Nanning, Guangxi 530003 P.R. China

**Keywords:** nonlinear equations, large-scale, conjugate gradient, quasi-Newton method, global convergence

## Abstract

In this paper, the algorithm for large-scale nonlinear equations is designed by the following steps: (i) a conjugate gradient (CG) algorithm is designed as a sub-algorithm to obtain the initial points of the main algorithm, where the sub-algorithm’s initial point does not have any restrictions; (ii) a quasi-Newton algorithm with the initial points given by sub-algorithm is defined as main algorithm, where a new nonmonotone line search technique is presented to get the step length $\alpha_{k}$. The given nonmonotone line search technique can avoid computing the Jacobian matrix. The global convergence and the $1+q$-order convergent rate of the main algorithm are established under suitable conditions. Numerical results show that the proposed method is competitive with a similar method for large-scale problems.

## Introduction

Consider the following nonlinear equations:

1.1 $$ e(x)=0,\quad x \in\Re^{n}. $$

Here $e:\Re^{n} \rightarrow\Re^{n}$ is continuously differentiable and *n* denotes large-scale dimension. The large-scale nonlinear equations are difficult to solve since the relations of the variables *x* are complex and the dimension is larger. Problem (1.1) can model many real-life problems, such as engineering problems, dimensions of mechanical linkages, concentrations of chemical species, cross-sectional properties of structural elements, etc. If the Jacobian $\nabla e(x)$ of *e* is symmetric, then problem (1.1) is called a system of symmetric nonlinear equations. Let *p* be the norm function with $p(x)=\frac{1}{2}\Vert e(x)\Vert ^{2}$, where $\Vert \cdot \Vert $ is the Euclidean norm. Then (1.1) is equivalent to the following global optimization models:

1.2 $$ \min p(x),\quad x\in\Re^{n}. $$

In fact, there are many actual problems that can convert to the above problems (1.2) (see [1–9] etc.) and have similar models (see [10–27] etc.). The iterative formula for (1.1) is

$$x_{k+1}=x_{k}+\alpha_{k} d_{k}, $$

where $\alpha_{k}$ is a step length and $d_{k}$ is a search direction. Now let us review some methods for $\alpha_{k}$ and $d_{k}$, respectively:

(i) Li and Fukashima [28] proposed an approximately monotone technique for $\alpha_{k}$:

1.3 $$ p(x_{k}+\alpha_{k}d_{k})-p(x_{k}) \leq -\delta_{1}\Vert \alpha_{k}d_{k}\Vert ^{2}-\delta_{2}\Vert \alpha_{k}e_{k} \Vert ^{2}+\epsilon_{k}\Vert e_{k}\Vert ^{2}, $$

where $e_{k}=e(x_{k})$, $\delta_{1}>0, \delta_{2}>0$ are positive constants, $\alpha_{k}=r^{i_{k}}, r\in(0,1), i_{k}$ is the smallest nonnegative integer *i* satisfying (1.3) and $\epsilon_{k}$ such that

1.4 $$ \sum_{k=0}^{\infty}\epsilon_{k}< \infty. $$

(ii) Gu *et al.* [29] presented a descent line search technique:

1.5 $$ p(x_{k}+\alpha_{k}d_{k})-p(x_{k}) \leq -\delta_{1}\Vert \alpha_{k}d_{k}\Vert ^{2}-\delta_{2}\Vert \alpha_{k}e_{k} \Vert ^{2}. $$

(iii) Brown and Saad [30] gave the following technique to obtain $\alpha_{k}$:

1.6 $$ p(x_{k}+\alpha_{k}d_{k})-p(x_{k}) \leq\beta\alpha_{k} \nabla p(x_{k})^{T}d_{k}, $$

where $\beta\in(0,1)$ and $\nabla p(x_{k})=\nabla e(x_{k}) e(x_{k})$.

(iv) Based on this technique, Zhu [31] proposed a nonmonotone technique:

1.7 $$ p(x_{k}+\alpha_{k}d_{k})-p(x_{l(k)}) \leq\beta\alpha_{k} \nabla p(x_{k})^{T}d_{k}, $$

$p(x_{l(k)})=\max_{0\leq j\leq m(k)}\{p(x_{k-j})\}$, $m(0)=0$, and $m(k)= \min\{m(k-1)+1,M\}$, $k\geq 1$, and *M* is a nonnegative integer.

(v) Yuan and Lu [32] gave a new technique:

1.8 $$ p(x_{k}+\alpha_{k}d_{k})-p(x_{k}) \leq\beta\alpha_{k}^{2} e(x_{k})^{T}d_{k}, $$

and some convergence results are obtained.

Next we present some techniques for the calculation of $d_{k}$. At present, there exist many well-known methods for $d_{k}$, such as the Newton method, the trust region method, and the quasi-Newton method, etc.

(i) The Newton method has the following form to get $d_{k}$:

1.9 $$ \nabla e(x_{k}) d_{k}=-e(x_{k}). $$

This method is regarded as one of the most effective methods. However, its efficiency largely depends on the possibility to efficiently solve (1.9) at each iteration. Moreover, the exact solution of the system (1.9) could be too burdensome when the iterative point $x_{k}$ is far from the exact solution [33]. In order to overcome this drawback, inexact quasi-Newton methods are often used.

(ii) The quasi-Newton method is of the form

1.10 $$ B_{k}d_{k}+e_{k}=0, $$

where $B_{k}$ is generated by a quasi-Newton update formula, where the BFGS (Broyden-Fletcher-Goldfarb-Shanno) update formula is one of the well-known quasi-Newton formulas with

1.11 $$ B_{k+1}=B_{k} - \frac{B_{k} s_{k} s_{k}^{T} B_{k}}{s_{k}^{T} B_{k} s_{k}} + \frac{y_{k} {y_{k}}^{T}}{{y_{k}}^{T} s_{k}}, $$

where $s_{k}=x_{k+1}-x_{k}, y_{k}=e_{k+1}-e_{k}$, and $e_{k+1}=e(x_{k+1})$. Set $H_{k}$ to be the inverse of $B_{k}$, then the inverse formula of (1.11) has the following form:

1.12 $$\begin{aligned} H_{k+1} =&H_{k}-\frac{{y_{k}}^{T}(s_{k}-H_{k}y_{k})s_{k}s_{k}^{T}}{({y_{k}}^{T}s_{k})^{2}} + \frac{(s_{k}-H_{k}y_{k})s_{k}^{T}+s_{k}(s_{k}-H_{k}y_{k})^{T}}{({y_{k}}^{T}s_{k})^{2}} \\ =& \biggl(I-\frac{s_{k}{y_{k}}^{T}}{{y_{k}}^{T}s_{k}} \biggr)H_{k} \biggl(I- \frac {y_{k}s_{k}^{T}}{{y_{k}}^{T}s_{k}} \biggr)+\frac{s_{k}s_{k}^{T}}{{y_{k}}^{T}s_{k}}. \end{aligned}$$

There exist many quasi-Newton methods (see [31, 32, 34–39]) representing the basic approach underlying most of the Newton-type large-scale algorithms.

The earliest nonmonotone line search framework was developed by Grippo, Lampariello, and Lucidi in [40] for Newton’s methods. Many subsequent papers have exploited nonmonotone line search techniques of this nature (see [41–44] etc.), which shows that the nonmonotone technique works well in many cases. Considering these points, Zhu [31] proposed the nonmonotone line search (1.7). From (1.7), we can see that the Jacobian matrix $\nabla e(x)$ must be computed at every iteration. Computing the Jacobian matrix $\nabla e(x)$ may be expensive if *n* is large and for any *n* at every iteration. Thus, one might prefer to remove the matrix, leading to a new nonmonotone technique.

Inspired by the above observations, we make a study of inexact quasi-Newton methods with a new nonmonotone technique for solving smooth nonlinear equations. In the *k*th iteration of our algorithm, the following new nonmonotone technique is used to obtain $\alpha_{k}$:

1.13 $$ p(x_{k}+\alpha_{k}d_{k})\leq p(x_{l(k)}) +\alpha_{k} \sigma e(x_{k})^{T}d_{k}, $$

where $\sigma\in(0,1)$ is a constant and $d_{k}$ is a solution of (1.10). Comparing with (1.7), the new technique (1.13) does not compute the Jacobian matrix $\nabla e(x)$. Then the storage and workload can be saved in theory. In Section 3, we will state the technique (1.13) is well defined.

It is well known that the initial point plays an important role in an algorithm. For example, the local superlinear convergence needs the iteration point *x* lies in the neighborhood of the optimal solution $x^{*}$, if the choice of the point *x* is correct then the Newton method can get the optimal solution $x^{*}$ just need one step, moreover, the correct initial point can speed up the efficiency of an algorithm. The nonlinear conjugate gradient method is one of the most effective line search methods for unconstrained optimization problems due to its simplicity and low memory requirement, especially for large-scale problems. Many scholars have made many studies and obtained lots of achievements on the CG methods or other similar new methods (see [45–49] etc.), where the results of [46] are especially interesting. It has been proved that the numerical performance of the CG methods is very interesting for large-scale problems in different application fields. These considerations prompt us to design a CG algorithm (sub-algorithm) for solving large-scale nonlinear equations, where the terminated iteration point of the CG algorithm was used as the initial point of the given algorithm (main algorithm). Then there exist two advantages from this process: one is that we can use the CG’s characteristic to get a better initial point and another is that the good convergent results of the main algorithm can be preserved. The main attributes of this paper are stated as follows:

- A sub-algorithm is designed to get the initial point of the main algorithm.
- A new nonmonotone line search technique is presented, moreover, the Jacobian matrix $\nabla e_{k}$ must not be computed at every iteration.
- The given method possesses the sufficient descent property for the normal function $p(x)$.
- The global convergence and the $1+q$-order convergent rate of the new method are established under suitable conditions.
- Numerical results show that this method is more effective than other similar methods.

We organize the paper as follows. In Section 2, the algorithms are stated. Convergent results are established in Section 3 and numerical results are reported in Section 4. In the last section, our conclusion is given. Throughout this paper, we use these notations: $\Vert \cdot \Vert $ is the Euclidean norm, $e(x_{k})$ and $g(x_{k+1})$ are replaced by $e_{k}$ and $g_{k+1}$, respectively.

## Algorithm

In this section, we will design a sub-algorithm and the main algorithm, respectively. These two algorithms are listed as follows.

**Initial point algorithm (sub-algorithm)**

Step 0: Given any $x_{0} \in\Re^{n}, \delta_{1},\delta_{2} \in(0,1), \epsilon_{k}>0$, $r \in(0,1), \epsilon\in[0,1)$, let $k:=0$.
Step 1: If $\Vert e_{k}\Vert \leq\epsilon$, stop. Otherwise let $d_{k}=-e_{k}$ and go to next step.
Step 2: Choose $\epsilon_{k+1}$ satisfies (1.4) and let $\alpha_{k}=1,r,r^{2},r^{3},\ldots $ until (1.3) holds.
Step 3: Let $x_{k+1}=x_{k} +\alpha_{k} d_{k}$.
Step 4: If $\Vert e_{k+1}\Vert \leq\epsilon$, stop.
Step 5: Compute $d_{k+1}=-e_{k+1}+\beta_{k}d_{k}$, set $k=k+1$ and go to Step 2.

### Remark

(i) $\beta_{k}$ of Step 5 is a scalar and different $\beta_{k}$ will determine different CG methods.

(ii) From Step 2 and [28], it is easy to deduce that there exists $\alpha_{k}$ such that (1.3). Thus, this sub-algorithm is well defined.

In the following, we will state the main algorithm. First, assume that the terminated point of sub-algorithm is $x_{sup}$, then the given algorithm is defined as follows.

### Algorithm 1

Main algorithm

*Step* 0: *Choose* $x_{sup} \in\Re^{n}$ *as the initial point*, *an initial symmetric positive definite matrix* $B_{0}\in\Re^{n\times n}$, *and constants* $r, \sigma\in(0,1), \epsilon_{\mathrm{main}}<\epsilon$, *a positive integer* $M>0$, $m(k)=0$, *let* $k:=0$;

Step 1: *Stop if* $\Vert e_{k}\Vert \leq\epsilon_{\mathrm{main}}$. *Otherwise solve* (1.10) *to get* $d_{k}$.
Step 2: *Let* $\alpha_{k}=1,r,r^{2},r^{3},\ldots $ *until* (1.13) *holds*.
Step 3: *Let the next iterative be* $x_{k+1}=x_{k}+\alpha_{k}d_{k}$.
Step 4: *Update* $B_{k}$ *by quasi*-*Newton update formula and ensure the update matrix* $B_{k+1}$ *is positive definite*.
Step 5: *Let* $k:=k+1$. *Go to Step* 1.

### Remark

Step 4 of Algorithm 1 can ensure that $B_{k}$ is always positive definite. This means that (1.10) has a unique solution $d_{k}$. By positive definiteness of $B_{k}$, it is easy to obtain $e_{k}^{T}d_{k}<0$. In the following sections, we only concentrate to the convergence of the main algorithm.

## Convergence analysis

Let Ω be the level set with

3.1 $$ \Omega= \bigl\{ x| \bigl\Vert e(x) \bigr\Vert \le \bigl\Vert e(x_{0}) \bigr\Vert \bigr\} . $$

Similar to [31, 32, 50], the following assumptions are needed to prove the global convergence of Algorithm 1.

### Assumption A

(i) *e* is continuously differentiable on an open convex set $\Omega_{1}$ containing Ω.

(ii) The Jacobian of *e* is symmetric, bounded, and positive definite on $\Omega_{1}$, *i.e.*, there exist positive constants $M^{*}\geq m_{*}>0$ such that

3.2 $$ \bigl\Vert \nabla e(x) \bigr\Vert \le M^{*} \quad \forall x \in \Omega_{1} $$

and

3.3 $$ m_{*}\Vert d\Vert ^{2}\le d^{T}\nabla e(x)d\quad \forall x \in\Omega_{1},d\in \Re^{n}. $$

### Assumption B

$B_{k}$ is a good approximation to $\nabla e_{k}$, *i.e.*,

3.4 $$ \bigl\Vert (\nabla e_{k}-B_{k})d_{k} \bigr\Vert \leq\epsilon_{*}\Vert e_{k}\Vert , $$

where $\epsilon_{*}\in(0,1)$ is a small quantity.

Considering Assumption B and using the von Neumann lemma, we deduce that $B_{k}$ is also bounded (see [31]).

### Lemma 3.1

*Let Assumption* B *hold*. *Then* $d_{k}$ *is a descent direction of* $p(x)$ *at* $x_{k}$, *i*.*e*.,

3.5 $$ \nabla p(x_{k})^{T}d_{k}\leq-(1- \epsilon_{*}) \bigl\Vert e(x_{k}) \bigr\Vert ^{2}. $$

### Proof

By using (1.10), we get

3.6 $$\begin{aligned} \nabla p(x_{k})^{T}d_{k} =& e(x_{k})^{T} \nabla e(x_{k}) d_{k} \\ =&e(x_{k})^{T} \bigl[ \bigl(\nabla e(x_{k})-B_{k} \bigr)d_{k}-e(x_{k}) \bigr] \\ =&e(x_{k})^{T} \bigl(\nabla e(x_{k})-B_{k} \bigr)d_{k} - e(x_{k})^{T}e(x_{k}). \end{aligned}$$

Thus, we have

$$\begin{aligned} \nabla p(x_{k})^{T}d_{k}+ \bigl\Vert e(x_{k}) \bigr\Vert ^{2} =&e(x_{k})^{T} \bigl(\nabla e(x_{k})-B_{k} \bigr)d_{k} \\ \leq& \bigl\Vert e(x_{k}) \bigr\Vert \bigl\Vert \bigl(\nabla e(x_{k})-B_{k} \bigr)d_{k} \bigr\Vert . \end{aligned}$$

It follows from (3.4) that

3.7 $$\begin{aligned} \nabla p(x_{k})^{T}d_{k} \leq& \bigl\Vert e(x_{k}) \bigr\Vert \bigl\Vert \bigl(\nabla e(x_{k})-B_{k} \bigr)d_{k} \bigr\Vert - \bigl\Vert e(x_{k}) \bigr\Vert ^{2} \\ \leq& -(1-\epsilon_{*}) \bigl\Vert e(x_{k}) \bigr\Vert ^{2}. \end{aligned}$$

The proof is complete. □

The following lemma shows that the line search technique (1.13) is reasonable, then Algorithm 1 is well defined.

### Lemma 3.2

*Let Assumptions* A *and* B *hold*. *Then Algorithm* 1 *will produce an iteration* $x_{k+1}=x_{k}+\alpha_{k}d_{k}$ *in a finite number of backtracking steps*.

### Proof

From Lemma 3.5 in [32] we have in a finite number of backtracking steps

$$p(x_{k}+\alpha_{k}d_{k})\leq p(x_{k}) +\alpha_{k} \sigma e(x_{k})^{T}d_{k}, $$

from which, in view of the definition of $p(x_{l(k)})=\max_{0\leq j\leq m(k)}\{p(x_{k-j})\}\geq p(x_{k})$, we obtain (1.13). Thus we conclude the result of this lemma. The proof is complete. □

Now we establish the global convergence theorem of Algorithm 1.

### Theorem 3.1

*Let Assumptions* A *and* B *hold*, *and* $\{\alpha_{k}, d_{k}, x_{k+1}, e_{k+1}\}$ *be generated by Algorithm* 1. *Then*

3.8 $$ \lim_{k\rightarrow\infty} \Vert e_{k}\Vert =0. $$

### Proof

By the acceptance rule (1.13), we have

3.9 $$ p(x_{k+1})-p(x_{l(k)}) \leq\sigma \alpha_{k} e_{k}^{T}d_{k} < 0. $$

Using $m(k+1)\leq m(k)+1$ and $p(x_{k+1})\leq p(x_{l(k)})$, we obtain

$$p(x_{l(k+1)})\leq\max \bigl\{ p(x_{l(k)}),p(x_{k+1}) \bigr\} =p(x_{l(k)}). $$

This means that the sequence $\{p(x_{l(k)})\}$ is decreasing for all *k*. Then $\{p(x_{l(k)})\}$ is convergent. Based on Assumptions A and B, similar to Lemma 3.4 in [32], it is not difficult to deduce that there exist constants $b_{1}\geq b_{2}>0$ such that

3.10 $$ b_{2}\Vert d_{k}\Vert ^{2}\leq d_{k}^{T}B_{k}d_{k}=-e_{k}^{T}d_{k} \leq b_{1}\Vert d_{k}\Vert ^{2}. $$

By (1.13) and (3.10), for all $k>M$, we get

3.11 $$\begin{aligned} p(x_{l(k)}) =&p(x_{l(k)-1}+\alpha_{l(k)-1}d_{l(k)-1}) \\ \leq&\max_{0\leq j\leq m(l(k)-1)} \bigl\{ p(x_{l(k)-j-1}) \bigr\} +\sigma \alpha_{l(k)-1}g_{l(k)-1}^{T}d_{l(k)-1} \\ \leq& \max_{0\leq j\leq m(l(k)-1)} \bigl\{ p(x_{l(k)-j-1}) \bigr\} -\sigma b_{2} \alpha_{l(k)-1}\Vert d_{l(k)-1}\Vert ^{2}. \end{aligned}$$

Since $\{p(x_{l(k)})\}$ is convergent, from the above inequality, we have

$$\lim_{k\rightarrow\infty}\alpha_{l(k)-1}\Vert d_{l(k)-1}\Vert ^{2}=0. $$

This implies that either

3.12 $$ \lim_{k\rightarrow\infty}\inf d_{l(k)-1}=0 $$

or

3.13 $$ \lim_{k\rightarrow\infty}\inf \alpha_{l(k)-1}=0. $$

If (3.12) holds, following [40], by induction we can prove that

3.14 $$ \lim_{k\rightarrow\infty} \Vert d_{l(k)-j}\Vert =0 $$

and

$$\lim_{k\rightarrow\infty} p(x_{l(k)-j})=\lim_{k\rightarrow\infty} p(x_{l(k)}) $$

for any positive integer *j*. As $k\geq l(k)\geq k-M$ and *M* is a positive constant, by

$$x_{k}=x_{k-M-1}+\alpha_{k-M-1}d_{k-M-1}+ \cdots+ \alpha_{l(k)-1}d_{l(k)-1} $$

and (3.14), it can be derived that

3.15 $$ \lim_{k\rightarrow\infty} p(x_{l(k)})=\lim _{k\rightarrow \infty} p(x_{k}). $$

According to (3.10) and the rule for accepting the step $\alpha_{k}d_{k}$,

3.16 $$ p(x_{k+1})-p(x_{l(k)})\leq\alpha_{k} \sigma e_{k}^{T}d_{k}\leq \alpha_{k} \sigma b_{2}\Vert d_{k}\Vert ^{2}. $$

This means

$$\lim_{k\rightarrow\infty}\alpha_{k}\Vert d_{k}\Vert ^{2}=0, $$

which implies that

3.17 $$ \lim_{k\rightarrow\infty}\alpha_{k}=0 $$

or

3.18 $$ \lim_{k\rightarrow\infty} \Vert d_{k}\Vert =0. $$

If equation (3.18) holds, since $B_{k}$ is bounded, then $\Vert e_{k}\Vert =\Vert B_{k}d_{k}\Vert \leq \Vert B_{k}\Vert \Vert d_{k}\Vert \rightarrow0$ holds. The conclusion of this lemma holds. If (3.17) holds. Then acceptance rule (1.13) means that, for all large enough *k*, $\alpha_{k}'=\frac{\alpha_{k}}{r}$ such that

3.19 $$\begin{aligned} p \bigl(x_{k}+\alpha_{k}'d_{k} \bigr)-p(x_{k}) \geq& p \bigl(x_{k}+\alpha_{k}'d_{k} \bigr)-p(x_{l(k)}) >\sigma\alpha_{k}'e_{k}^{T}d_{k}. \end{aligned}$$

Since

3.20 $$ p \bigl(x_{k}+\alpha_{k}'d_{k} \bigr)-p(x_{k})= \alpha_{k}' \nabla p(x_{k})^{T}d_{k}+o \bigl(\alpha_{k}' \Vert d_{k}\Vert \bigr). $$

Using (3.19) and (3.20) in [32], we have

$$\nabla p(x_{k})^{T}d_{k}=e_{k}^{T} \nabla e(x_{k})d_{k}\leq\delta^{*} e_{k}^{T}d_{k}, $$

where $\delta^{*} >0$ is a constant and $\sigma< \delta^{*}$. So we get

3.21 $$ \bigl[\delta^{*}-\sigma \bigr] \alpha_{k}' e_{k}^{T}d_{k}+o \bigl(\alpha_{k}' \Vert d_{k}\Vert \bigr)\geq0. $$

Note that $\delta^{*}-\sigma>0$ and $e_{k}^{T}d_{k}<0$, we have from dividing (3.21) by $\alpha_{k}'\Vert d_{k}\Vert $

3.22 $$ \lim_{k\rightarrow\infty}\frac{e_{k}^{T}d_{k}}{\Vert d_{k}\Vert }=0. $$

By (3.10), we have

3.23 $$ \lim_{k\rightarrow\infty} \Vert d_{k}\Vert =0. $$

Consider $\Vert e_{k}\Vert =\Vert B_{k}d_{k}\Vert \leq \Vert B_{k}\Vert \Vert d_{k}\Vert $ and the bounded $B_{k}$ again, we complete the proof. □

### Lemma 3.3

see Lemma 4.1 in [31]

*Let* *e* *be continuously differentiable*, *and* $\nabla e(x)$ *be nonsingular at* $x^{*}$ *which satisfies* $e(x^{*})=0$. *Let*

3.24 $$ a\equiv \biggl\{ \bigl\Vert \nabla e \bigl(x^{*} \bigr) \bigr\Vert + \frac{1}{2c},2c \biggr\} , c= \bigl\Vert \nabla e \bigl(x^{*} \bigr)^{-1} \bigr\Vert . $$

*If* $\Vert x_{k}-x^{*}\Vert $ *sufficiently small*, *then the inequality*

3.25 $$ \frac{1}{a} \bigl\Vert x_{k}-x^{*} \bigr\Vert \leq \bigl\Vert e(x_{k}) \bigr\Vert \leq a \bigl\Vert x_{k}-x^{*} \bigr\Vert $$

*holds*.

### Theorem 3.2

*Let the assumptions in Lemma* 3.3 *hold*. *Assume that there exists a sufficiently small* $\varepsilon_{0}>0$ *such that* $\Vert B_{k}-\nabla e(x_{k})\Vert \leq\varepsilon_{0}$ *for each* *k*. *Then the sequence* $\{x_{k}\}$ *converges to* $x^{*}$ *superlinearly for* $\alpha_{k}=1$. *Moreover*, *if* *e* *is q*-*order smooth at* $x^{*}$ *and there is a neighborhood* *U* *of* $x^{*}$ *satisfying for any* $x_{k}\in U$,

3.26 $$ \bigl\Vert \bigl[B_{k}-\nabla e \bigl(x^{*} \bigr) \bigr] \bigl(x_{k}-x^{*} \bigr) \bigr\Vert \leq\eta \bigl\Vert x_{k}-x^{*} \bigr\Vert ^{1+q}, $$

*then* $x_{k}\rightarrow x^{*}$ *with order at least* $1+q$, *where* *η* *is a constant*.

### Proof

Since *g* is continuously differentiable and $\nabla e(x)$ is nonsingular at $x^{*}$, there exists a constant $\gamma>0$ and a neighborhood *U* of $x^{*}$ satisfying

$$\max \bigl\{ \bigl\Vert \nabla e(y) \bigr\Vert , \bigl\Vert \nabla e(y)^{-1} \bigr\Vert \bigr\} \leq\gamma, $$

where $\nabla e(y)$ is nonsingular for any $y\in U$. Consider the following equality when $\alpha_{k}=1$:

3.27 $$\begin{aligned} &B_{k} \bigl(x_{k+1}-x^{*} \bigr)+ \bigl[ \nabla e(x_{k}) \bigl(x_{k}-x^{*} \bigr)-B_{k} \bigl(x_{k}-x^{*} \bigr) \bigr] \\ &\qquad{}+ \bigl[e(x_{k})-e \bigl(x^{*} \bigr)-\nabla e(x_{k}) \bigl(x_{k}-x^{*} \bigr) \bigr] \\ &\quad=e(x_{k})+B_{k}d_{k}=0, \end{aligned}$$

the second term and the third term are $o(\Vert x_{k}-x^{*}\Vert )$. By the von Neumann lemma, and considering that $\nabla e(x_{k})$ is nonsingular, $B_{k}$ is also nonsingular. For any $y\in U$ and $\nabla e(y)$ being nonsingular and $\max\{\Vert \nabla e(y)\Vert ,\Vert \nabla e(y)^{-1}\Vert \}\leq\gamma$, then we obtain from Lemma 3.3

$$\bigl\Vert x_{k+1}-x^{*} \bigr\Vert =o \bigl( \bigl\Vert x_{k}-x^{*} \bigr\Vert \bigr)=o \bigl( \bigl\Vert e(x_{k}) \bigr\Vert \bigr),\quad \mbox{as }k\rightarrow \infty, $$

this means that the sequence $\{x_{k}\}$ converges to $x^{*}$ superlinearly for $\alpha_{k}=1$.

If *e* is *q*-order smooth at $x^{*}$, then we get

$$e(x_{k})-e \bigl(x^{*} \bigr)-\nabla e(x_{k}) \bigl(x_{k}-x^{*} \bigr)=O \bigl( \bigl\Vert x_{k}-x^{*} \bigr\Vert ^{q+1} \bigr). $$

Consider the second term of (3.27) as $x_{k}\rightarrow x^{*}$, and use (3.26), we can deduce that the second term of (3.27) is also $O(\Vert x_{k}-x^{*}\Vert ^{q+1})$. Therefore, we have

$$\bigl\Vert x_{k+1}-x^{*} \bigr\Vert =O \bigl( \bigl\Vert x_{k}-x^{*} \bigr\Vert ^{q+1} \bigr), \quad\mbox{as }x_{k} \rightarrow x^{*}. $$

The proof is complete. □

## Numerical results

In this section, we report results of some numerical experiments with the proposed method. The test functions have the following form:

$$e(x)= \bigl(f_{1}(x),f_{2}(x),\ldots,f_{n}(x) \bigr)^{T}, $$

where these functions have the associated initial guess $x_{0}$. These functions are stated as follows.

### Function 1

Exponential function 2

$$\begin{aligned} f_{1}(x) =&e^{x_{1}}-1, \\ f_{i}(x) =&\frac{i}{10} \bigl(e^{x_{i}}+x_{i-1}-1 \bigr),\quad i=2,3,\ldots,n. \end{aligned}$$

Initial guess: $x_{0}=(\frac{1}{n^{2}},\frac{1}{n^{2}},\ldots,\frac{1}{n^{2}})^{T}$.

### Function 2

Trigonometric function

$$f_{i}(x)=2 \Biggl(n+i(1-\cos x_{i})-\sin x_{i}- \sum_{j=1}^{n} \cos x_{j} \Biggr) (2\sin x_{i}-\cos x_{i}),\quad i=1,2,3,\ldots,n. $$

Initial guess: $x_{0}=(\frac{101}{100n},\frac{101}{100n},\ldots,\frac {101}{100n})^{T}$.

### Function 3

Logarithmic function

$$f_{i}(x)=\ln(x_{i}+1)-\frac{x_{i}}{n},\quad i=1,2,3, \ldots,n. $$

Initial guess: $x_{0}=(1,1,\ldots,1)^{T}$.

### Function 4

Broyden tridiagonal function [[51], pp. 471-472]

$$\begin{aligned} f_{1}(x) =&(3-0.5x_{1})x_{1}-2x_{2}+1, \\ f_{i}(x) =&(3-0.5x_{i})x_{i}-x_{i-1}+2x_{i+1}+1, \\ & i=2,3,\ldots,n-1, \\ f_{n}(x) =&(3-0.5x_{n})x_{n}-x_{n-1}+1. \end{aligned}$$

Initial guess: $x_{0}=(-1,-1,\ldots,-1)^{T}$.

### Function 5

Trigexp function [[51], p. 473]

$$\begin{aligned} f_{1}(x) =&3x_{1}^{3}+2x_{2}-5+ \sin(x_{1}-x_{2})\sin(x_{1}+x_{2}), \\ f_{i}(x) =&-x_{i-1}e^{x_{i-1}-x_{i}}+x_{i} \bigl(4+3x_{i}^{2} \bigr)+2x_{i+1} \\ &{} +\sin(x_{i}-x_{i+1})\sin(x_{i}+x_{i+1})-8,\quad i=2,3,\ldots,n-1, \\ f_{n}(x) =&-x_{n-1}e^{x_{n-1}-x_{n}}+4x_{n}-3. \end{aligned}$$

Initial guess: $x_{0}=(0,0,\ldots,0)^{T}$.

### Function 6

Strictly convex function 1 [[52], p. 29]. $e(x)$ is the gradient of $h(x)=\sum_{i=1}^{n}(e^{x_{i}}-x_{i})$.

$$f_{i}(x)=e^{x_{i}}-1,\quad i=1,2,3,\ldots,n. $$

Initial guess: $x_{0}=(\frac{1}{n},\frac{2}{n},\ldots,1)^{T}$.

### Function 7

Strictly convex function 2 [[52], p. 30]

$e(x)$ is the gradient of $h(x)=\sum_{i=1}^{n}\frac{i}{10}(e^{x_{i}}-x_{i})$.

$$f_{i}(x)=\frac{i}{10} \bigl(e^{x_{i}}-1 \bigr),\quad i=1,2,3, \ldots,n. $$

Initial guess: $x_{0}=(1,1,\ldots,1)^{T}$.

### Function 8

Variable dimensioned function

$$\begin{aligned} f_{i}(x) =&x_{i}-1,\quad i=1,2,3,\ldots,n-2, \\ f_{n-1}(x) =& \sum_{j=1}^{n-2}j(x_{j}-1), \\ f_{n}(x) =& \Biggl(\sum_{j=1}^{n-2}j(x_{j}-1) \Biggr)^{2}. \end{aligned}$$

Initial guess: $x_{0}=(1-\frac{1}{n},1-\frac{2}{n},\ldots,0)^{T}$.

### Function 9

Discrete boundary value problem [53].

$$\begin{aligned} f_{1}(x) =&2x_{1}+0.5h^{2}(x_{1}+h)^{3}-x_{2}, \\ f_{i}(x) =&2x_{i}+0.5h^{2}(x_{i}+hi)^{3}-x_{i-1}+x_{i+1}, \\ &i=2,3,\ldots,n-1 \\ f_{n}(x) =&2x_{n}+0.5h^{2}(x_{n}+hn)^{3}-x_{n-1}, \\ h =&\frac{1}{n+1}. \end{aligned}$$

Initial guess: $x_{0}=(h(h-1),h(2h-1),\ldots,h(nh-1))$.

### Function 10

The discretized two-point boundary value problem similar to the problem in [53]

$$e(x)= Ax+\frac{1}{(n+1)^{2}}F(x)=0, $$

when *A* is the $n\times n$ tridiagonal matrix given by

$$A=\left [ \begin{matrix}8 & -1 \\ -1 & 8 & -1\\ & -1 & 8 & -1 \\ &&\ddots& \ddots& \ddots& \\ &&&\ddots& \ddots& -1 \\ &&&& -1 & 8 \end{matrix} \right ], $$

and $F(x)=(F_{1}(x),F_{2}(x),\ldots,F_{n}(x))^{T}$ with $F_{i}(x)=\sin x_{i} -1, i=1,2,\ldots,n$, and $x_{0}=(50,0, 50,0,\ldots)$.

In the experiments, all codes were written in MATLAB r2009a and run on a PC with G1620T@2.40 GHz CPU processor and 4.0 GB memory and Windows XP operation system. In order to compare the performance the given algorithm with CG’s initial points (called new method with CG), we also do the experiment with only the main algorithm with initial points $x_{0}$ (called the normal method). Aslam Noor *et al.* [54] presented a variational iteration technique for nonlinear equations, where the so-called VIM1 method has the better numerical performance. The VIM1 method has the following iteration form:

$$x_{k+1}=x_{k}- \bigl[\nabla e-\mathbf{diag}( \beta_{1}e_{1},\beta_{2}e_{2},\ldots , \beta _{n}e_{n}) \bigr]^{-1}(x_{k})e(x_{k}), $$

where $\beta_{i}\in(0,1)$ for $i=1,2,\ldots,n$. In their paper, only low dimension problems (two variables) are tested. In this experiment, we also give the numerical results of this method for large-scale nonlinear equations to compare with our proposed algorithm.

The parameters were chosen as $r=0.1$, $\sigma=0.9, M=12,\epsilon=10^{-4}$, and $\epsilon_{\mathrm{main}}=10^{-5}$. In order to ensure the positive definiteness of $B_{k}$, in Step 4 of the main algorithm: if $y_{k}^{T}s_{k}>0$, update $B_{k}$ by (1.11), otherwise let $B_{k+1}=B_{k}$. This program will also be stopped if the iteration number of main algorithm is larger than 200. Since the line search cannot always ensure these descent conditions $d_{k}^{T}e_{k}<0$ and $d_{k}^{T}\nabla e(x_{k}) e_{k}<0$, an uphill search direction may occur in numerical experiments. In this case, the line search rule maybe fails. In order to avoid this case, the stepsize $\alpha_{k}$ will be accepted if the searching time is larger than six in the inner circle for the test problems.

In the sub-algorithm, the CG formula is used by the following Polak-Ribière-Polyak (PRP) method [55, 56]

4.1 $$ d_{k}= \textstyle\begin{cases} -e_{k}+\frac{e_{k}^{T}(e_{k}-e_{k-1})}{\Vert e_{k-1}\Vert ^{2}}d_{k-1} & \mbox{if } k\geq1,\\ -e_{k}& \mbox{if } k=0. \end{cases} $$

For the line search technique, (1.3) is used and the largest search number of times is ten, where $\delta_{1}=\delta_{2}=10^{-7}$, and $\epsilon_{k}=\frac{1}{NI^{2}}$ (*NI* is the iteration number). The sub-algorithm will also stopped if the iteration number is larger than 150. The iteration number, the function evaluations, and the CPU time of the sub-algorithm are added to the main algorithm for new method with CG. The meaning of the items of the columns of Table 1 is:

**Table 1:** **Numerical results**

		**New method with CG**				**Normal method (only main algorithm)**			
**P**	**Dim**	**NI/NG**	**GF**	**GD**	**cpu time**	**NI/NG**	**GF**	**GD**	**cpu time**
1	1000	1/1	6.676674e−006	1.335335e−005	0.000000e+000	0/2	6.676674e−006	1.335335e−005	0.000000e+000
	2000	1/1	3.335834e−006	6.671668e−006	0.000000e+000	0/2	3.335834e−006	6.671668e−006	0.000000e+000
	3000	1/1	2.223334e−006	4.446667e−006	1.560010e−002	0/2	2.223334e−006	4.446667e−006	3.120020e−002
2	1000	12/17	1.570352e−007	1.214954e−007	1.544410e+000	199/2879	1.624268e−004	1.228551e+000	1.338801e+002
	2000	200/2927	8.144022e−005	3.138945e−001	8.647135e+002	199/2928	8.144022e−005	3.138945e−001	8.680832e+002
	3000	200/2326	5.434381e−005	2.481121e−003	1.614626e+003	199/2327	5.434381e−005	2.481121e−003	1.622785e+003
3	1000	8/8	4.194859e−006	8.389718e−006	1.560010e−002	115/1009	5.838535e−008	1.171251e−007	7.996611e+001
	2000	8/8	7.775106e−006	1.555021e−005	7.800050e−002	117/1040	1.161670e−007	2.328056e−007	5.662368e+002
	3000	9/9	1.614597e−012	3.231630e−012	1.553770e+001	137/1362	1.739498e−007	3.484891e−007	2.141410e+003
4	1000	92/165	5.632576e−006	5.669442e−006	2.215214e+000	199/285	3.703283e+001	1.196051e+001	1.356897e+002
	2000	87/156	6.245922e−006	6.085043e−006	1.502290e+001	199/230	3.637504e+000	9.570779e+000	9.591097e+002
	3000	94/169	6.678153e−006	6.437585e−006	4.731510e+001	199/234	2.639260e+002	1.904865e+001	3.096277e+003
5	1000	22/51	8.288299e−006	6.268946e−007	2.106014e+000	199/2570	3.195300e+004	4.649782e+006	1.779971e+001
	2000	21/50	4.114462e−006	1.943351e−006	5.179233e+000	199/2652	6.395300e+004	1.354972e+005	8.327333e+001
	3000	21/51	9.843373e−006	8.504719e−006	1.597450e+001	199/2853	9.595300e+004	1.135356e+005	1.314776e+002
6	1000	9/11	5.984185e−012	1.197596e−011	7.176046e−001	6/9	6.069722e−007	1.118437e−006	4.149627e+000
	2000	9/11	1.505191e−006	3.010383e−006	7.800050e−002	6/9	1.210931e−006	2.231765e−006	2.898499e+001
	3000	9/11	2.251571e−006	4.503142e−006	1.404009e−001	6/9	1.814891e−006	3.345093e−006	9.300780e+001
7	1000	200/602	1.208240e−003	4.697005e+000	3.424222e+001	199/573	3.156137e+005	3.893380e+004	1.378113e+002
	2000	200/760	1.612671e+001	9.034319e+000	2.420200e+002	199/644	1.014481e+006	3.131576e+005	9.872367e+002
	3000	200/693	5.570227e−003	9.501149e+001	7.698181e+002	199/743	9.357473e+007	2.087488e+007	3.087119e+003
8	1000	2/2	0.000000e+000	0.000000e+000	0.000000e+000	1/3	0.000000e+000	0.000000e+000	6.552042e−001
	2000	2/2	0.000000e+000	0.000000e+000	0.000000e+000	1/3	0.000000e+000	0.000000e+000	4.820431e+000
	3000	2/2	0.000000e+000	0.000000e+000	6.240040e−002	1/3	0.000000e+000	0.000000e+000	1.538170e+001
9	1000	67/118	7.138941e−006	1.820053e−005	3.010819e+000	2/5	2.358640e−006	4.611203e−006	1.404009e+000
	2000	70/124	6.342724e−006	1.607326e−005	2.062333e+001	2/5	5.917002e−007	1.169969e−006	9.703262e+000
	3000	74/131	7.447187e−006	1.799920e−005	6.450641e+001	2/5	2.632811e−007	5.225655e−007	3.084140e+001
10	1000	26/49	2.044717e−008	3.900140e−008	2.359983e+002	121/125	7.382123e−006	1.467673e−005	4.987196e+002
	2000	24/47	9.030382e−006	2.717060e−006	1.847286e+003	121/125	7.454090e−006	1.481981e−005	3.852538e+003
	3000	27/51	6.468831e−009	1.138377e−008	6.632227e+003	121/125	7.523322e−006	1.495745e−005	1.299774e+004

Dim: the dimension.

NI: the number of iterations.

NG: the number of function evaluations.

cpu time: the cpu time in seconds.

GF: the final norm function evaluations $p(x)$ when the program is stopped.

GD: the final norm evaluations of search direction $d_{k}$.

fails: fails to find the final values of $p(x)$ when the program is stopped.

From Tables 1-2, it is easy to see that the number of iterations and the number of function evaluations of the new method with CG are less than those of the normal method for these test problems. Moreover, the cpu time and the final function norm evaluations of the new method with CG are more competitive than those of the normal method. For the VIM1 method, the results of Problems 1-7 are very interesting, but it fails for Problems 8-10. Moreover, it is not difficult to find that more CUP time is needed for this method. The main reason maybe lies in the computation of the Jacobian matrix at every iteration.

**Table 2:** **Numerical results of VIM1 method**

**P**	**Dim**	**NI/NG**	**GF**	**cpu time**	**P**	**Dim**	**NI/NG**	**GF**	**cpu time**
1	1000	1/1	6.676674e−006	1.560010e−002	6	1000	5/5	4.591162e−011	9.656462e+000
	2000	1/1	3.335834e−006	0.000000e+000		2000	5/5	9.140464e−011	7.439688e+001
	3000	1/1	2.223334e−006	3.120020e−002		3000	5/5	1.368978e−010	2.484628e+002
2	1000	18/18	2.840705e−007	5.494355e+001	7	1000	5/5	4.058902e−006	9.656462e+000
	2000	27/27	2.532474e−006	6.315544e+002		2000	6/6	1.983880e−017	8.993458e+001
	3000	22/22	9.781547e−007	1.669476e+003		3000	6/6	6.708054e−017	3.007543e+002
3	1000	5/5	5.430592e−007	9.578461e+000	8	1000	fails		
	2000	5/5	5.619751e−007	7.435008e+001		2000	fails		
	3000	5/5	5.870798e−007	2.484160e+002		3000	fails		
4	1000	4/4	4.559227e−009	1.243328e+001	9	1000	fails		
	2000	4/4	9.082664e−009	1.026487e+002		2000	fails		
	3000	4/4	1.360090e−008	3.708768e+002		3000	fails		
5	1000	9/9	2.648764e−006	3.196460e+001	10	1000	fails		
	2000	9/9	2.649263e−006	2.529244e+002		2000	fails		
	3000	9/9	2.649430e−006	8.258849e+002		3000	fails		

The tool of Dolan and Moré [57] is used to analyze the efficiency of these three algorithms.

**Figure 1:**
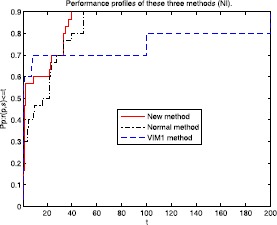
Performance profiles of these three methods (NI).

**Figure 2:**
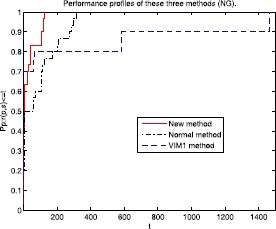
Performance profiles of these three methods (NG).

**Figure 3:**
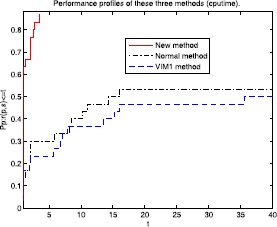
Performance profiles of these three methods (cpu time).

Figures 1-3 show that the performance of these methods are relative to NI, NG, and cpu time of Tables 1-2, respectively. The numerical results indicate that the proposed method performs best among these three methods. To this end, we think that the enhancement of this proposed method is noticeable.

## Conclusion

In this paper, we focus on two algorithms solved a class of large-scale nonlinear equations. At the first step, a CG algorithm, called a sub-algorithm, was used as the initial points of the main algorithm. Then a quasi-Newton algorithm with the initial points done by a CG sub-algorithm was defined as the main algorithm. In order to avoid computing the Jacobian matrix, a nonmonotone line search technique was used in the algorithms. The convergence results are established and numerical results are reported.

According to the numerical performance, it is clear that the CG technique is very effective for large-scale nonlinear equations. This observation inspires us to design the CG methods to directly solve nonlinear equations in the future.
